# A Machine-Learning-Based IoT System for Optimizing Nutrient Supply in Commercial Aquaponic Operations

**DOI:** 10.3390/s22093510

**Published:** 2022-05-05

**Authors:** Sambandh Bhusan Dhal, Kyle Jungbluth, Raymond Lin, Seyed Pouyan Sabahi, Muthukumar Bagavathiannan, Ulisses Braga-Neto, Stavros Kalafatis

**Affiliations:** 1 Department of Electrical and Computer Engineering, Texas A&M University, College Station, TX 79016, USA; sambandh@tamu.edu (S.B.D.); kjungbluth@tamu.edu (K.J.); rlin9264@tamu.edu (R.L.); pouyansabahi@tamu.edu (S.P.S.); ulisses@tamu.edu (U.B.-N.); 2 Department of Soil and Crop Sciences, Texas A&M University, College Station, TX 79016, USA; muthu.bagavathiannan@tamu.edu

**Keywords:** aquaponic, pairwise correlation matrix, XGBoost, Recursive Feature Elimination, ExtraTreesClassifier, median, closed loop

## Abstract

Nutrient regulation in aquaponic environments has been a topic of research for many years. Most studies have focused on appropriate control of nutrients in an aquaponic set-up, but very little research has been conducted on commercial-scale applications. In our model, the input data were sourced on a weekly basis from three commercial aquaponic farms in Southeast Texas over the course of a year. Due to the limited number of data points, dimensionality reduction techniques such as pairwise correlation matrix were used to remove the highly correlated predictors. Feature selection techniques such as the XGBoost classifier and Recursive Feature Elimination with ExtraTreesClassifier were used to rank the features in order of their relative importance. Ammonium and calcium were found to be the top two nutrient predictors, and based on the months in which lettuce was cultivated, the median of these nutrient values from the historical dataset served as the optimal concentration to be maintained in the aquaponic solution to sustain healthy growth of tilapia fish and lettuce plants in a coupled set-up. To accomplish this, Vernier sensors were used to measure the nutrient values and actuator systems were built to dispense the appropriate nutrient into the ecosystem via a closed loop.

## 1. Introduction

The system of aquaponics involves the adaptation of hydroponics and aquaculture in a combined set-up which can go on to eliminate the food scarcity and environmental crisis that the world faces today [1,2]. Due to rapid industrialization and large-scale globalization, the economic and social aspects of the varieties of food products emerging from aquaponics have been of great interest as research studies go on to show that it is possible to achieve a ten-fold increase in production without the use of harmful chemicals or pesticides, while using only 2–10% of the water used in traditional agricultural techniques [3,4,5]. Although there have been many studies over the last three decades on growing plants in aquaponic set-ups, none of them have focused specifically on determining the important nutrients which need to be monitored and controlled, depending on the season in which the crop was cultivated.

There have been numerous studies in which different Internet of Things (IoT) systems have been implemented in small laboratory set-ups for optimal growth of plants in hydroponic and aquaponic environments. In [6], Valiente et al. constructed a system to monitor the pH level and water temperature for growing Nile tilapia and romaine lettuce, and constructed an actuation system to maintain the pH level between 6.2 and 7.5 and water temperature between 27 °C and 30 °C to ensure optimal growth. In [7], Yanes et al. conducted a review study on IoT and smart systems for monitoring and regulating nitrite concentrations, electroconductivity, dissolved oxygen and hardness in the aquaponic solution to ensure a viable commercial model that can be implemented in small-scale semi-automated systems. In [8], Mahanta et al. performed a comprehensive comparative study on the growth of soybeans in hydroponic solution by applying different concentrations of plasma-activated water at varying voltages and time intervals to the seeds to minimize heavy metal uptake and ensure better yield. In [9,10,11,12], the process of sensing and controlling the general parameters of the aquaponic solution such as pH and water temperature for growing lettuce and other greens was carried out using different cloud-based approaches. However, very little has been done to formulate data-driven approaches to regulate the important nutrients for growing fish and plants; this serves as the main motivation behind this paper.

One of the major problems that one faces while designing an intelligent system for nutrient regulation in aquaponic systems is lack of data. For this, different synthetic data generation techniques have been used. In [13], Soltana et al. used an iterative approach to generate data samples that meet the desired statistical distribution, without taking into account the logical constraints, and later tweaking them to fix any violations. In [14], Anderson et al. proposed the generation of synthetic data for IoT devices which included extracting the structure of Extensible Markup Language (XML) files and characterizing the values within the dataset. In [15], Alzantot et al. presented a deep-learning-based architecture for synthesizing sensory data named Sensegen which comprised a Long Short-Term Memory (LSTM) network and a Mixture Density Network (MDN) in the first step, followed by an LSTM-network-based discriminator to differentiate between real and generated synthetic data. Similarly, in [16], Dahmen et al. presented an ML-based synthetic data generation technique named SynSys which comprises nested sequences using Markov models and regression models which are trained on real data. Similarly, in [17,18,19], the concept of synthetic data generation using Monte-Carlo approaches has been emphasized by generating a mean and covariance matrix between the classes.

The other problem which is generally faced while using small datasets is selection of relevant features from the dataset. In [20], Chandrashekar et al. conducted a survey of the feature selection methods which included preprocessing of the training data using Principal Component Analysis (PCA) and Fast Fourier Transform (FFT), selection of appropriate features using different filter, wrapper and embedded methods, and in the end, carrying out classification using Radial Basis Function (RBF), Support Vector Machine (SVM) and Artificial Neural Network (ANN) classifiers. In [21], Kira et al. focused on empirical test results in two artificial domains using the Light Emitting Diode (LED) Display domain and the Parity domain both with and without noise with a goal to optimize learning and improve quality. In [22], Li et al. proposed feature selection algorithms on conventional data by using similarity-based, theoretical-information-based, sparse-learning-based and statistical-based methods. In [23,24], the concept of correlation matrix to eliminate the highly correlated predictors was elaborated upon. In [25], an ensemble of various ML algorithms, namely, Decision Trees, Random Forest, ExtraTreesClassifier, Multi-Layer Perceptron (MLP) and Support Vector Machine (SVM) was used to generate Histogram of Oriented Gradients (HOG) features required for feature extraction. Similarly, in [26], Shafique et al. conducted a comparative study of different classification algorithms and ended up with a 90% accuracy score in detecting cardiovascular diseases using ExtraTreesClassifier. In [27,28,29,30], another important feature selection technique called XGBoost classifier has been implemented in healthcare to generate feature importance and reduce the size of the datasets, resulting in improved classification accuracy.

The regulation of parameters selected using the aforementioned feature selection techniques using an IoT-based set-up is of prime importance as it is an important step in automating the parameters needed for symbiotic growth of plants and fish in aquaponic systems. Some notable research in this area is described below. In [31], Rau et al. proposed a smart irrigation system consisting of a DHT11 temperature and humidity sensor and a Raspberry Pi to regularly monitor the weather conditions using an Android application for growing rice. Similarly, in [32], Zhang et al. proposed the use of sensors to measure air temperature, air humidity, soil temperature and soil humidity to monitor the growth of Chinese citrus in real-time environments using Zigbee technology. In [33], Yimwadsana et al. proposed an IoT-controlled system for plant growth consisting of sensors to measure air, light intensity and soil moisture, a dashboard to visualize the collected data and actuator modules consisting of relays, motor gear and water pump controlled by microcontrollers. In [34], Chaudhary et al. proposed a system consisting of a wireless sensor network to monitor and control the environmental parameters in a greenhouse. Similar to the above, in [35], Jaiswal et al. constructed a system to measure the parameters stated above in a greenhouse, controlling them through a Raspberry Pi module and displaying the data on a ThingSpeak cloud platform. Our research measures and regulates the important nutrients in the aquaponic solution which are determined from data-driven approaches rather than regulating the chemical properties of the solution such as pH and conductivity or the environmental parameters of the greenhouse such as temperature and humidity.

## 2. Methodology

**Figure 1:**
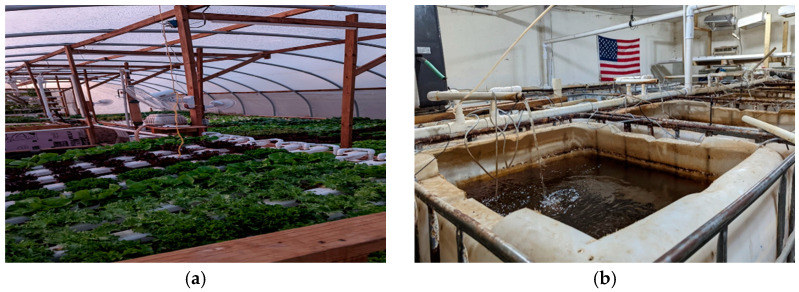
(**a**). set-up of the plant bed which was used for producing lettuce and other greens; and (**b**). a set-up of an array of interconnected fish tanks which served as a nutrient source for these plants in Texas US Farms, Grimes, TX, USA.

The dataset used in our case was recorded over the course of a year from three aquaponic facilities in Southeast Texas: Aquatic Greens Farm (Bryan), Wolff Family Farms (Caldwell) and Texas US Farms (Grimes). From the Aquatic Greens Farm, three samples were collected weekly (one from the tank which bred goldfish, another from the tank which bred tilapia and one from the greenhouse where lettuce was grown). From the Wolff Family Farms, two samples were collected weekly (one from the tank which bred Nile tilapia and the other from the main greenhouse where collard greens, jalapeno peppers and lettuce were grown). From the Texas US Farms, three samples were collected weekly (one from the main growth tank which was used to breed tilapia and shrimp, one from the greenhouse which was used to grow lettuce and kale greens and the other from the tank which was used to breed seedlings before placing them in the main plant bed). After collecting the samples, these were sent to the Soil, Forage and Water Testing Laboratory at Texas A&M University, USA for determining the major nutrients in the aquaponic solution. In the laboratory, the nitrate concentration in the aquaponic solution was measured by reducing the nitrites to nitrates using a cadmium column followed by spectrophotometry. The chloride concentration in the aquaponic solution was reduced by chromatography and the carbonate/bicarbonate concentration was determined by using acid titration using sulfuric acid. A set-up of the fish tank and the main plant bed which was used for growing lettuce is shown in Figure 1.

### 2.1. Analysis of the Dataset

The dataset which was used for analysis in our case had a total of 211 observations and 12 predictors. The predictors consisted of the concentrations of calcium, magnesium, sodium, potassium, boron, carbonate, bicarbonate, sulfate, chloride, nitrate, ammonium and phosphorus (all of these measured in ppm). The month in which the observations were recorded was used as the class variable, i.e., the winter and spring months from November to March in which plants were cultivated were coded as 1, and the summer and fall months from April to October were coded as 0, respectively. The predictor which stored the carbonate concentration in ppm was dropped before carrying out any preliminary analysis as the variance was zero throughout.

### 2.2. Generation of Synthetic Data

As the dataset was too small to make inferences on, it was an important measure to generate synthetic data before applying any data-driven approach. The dataset at hand had 107 observations belonging to Class 0 and 104 observations belonging to Class 1. Therefore, it was decided to use the concept of generation of mean and covariance matrix between and among the classes using two variations of Monte-Carlo (MC) approaches to generate synthetic data which were used for carrying out inferences.

At first, a mean vector and covariance matrix were generated between the observations belonging to class 0 and also for class 1. Both classes were considered separately while generating these matrices, and based on these computed parameters, the synthetic data points were generated. The same process was repeated again where all the observations were considered together as a single dataset, irrespective of their classes, to generate the mean vector and covariance matrices that were used to produce the second set of synthetic data. In the end, both datasets were combined and different feature selection techniques were carried out on them to select the top two macronutrient predictors, as described in detail in the next section.

### 2.3. Feature Selection

**Figure 2:**
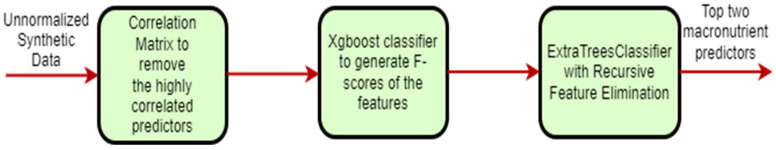
Feature selection pipeline for choice of appropriate nutrient predictors.

Before constructing an IoT system for sensing and regulating the nutrient parameters in a feedback loop, it was important to select the most important parameters to scale down the cost of the system. For this, three methods were used in a pipeline for reducing the dimensionality of the dataset, which is depicted in Figure 2 below.

The initial dataset which was used in this case followed a multivariate normal distribution, due to which, it was decided not to normalize the dataset before proceeding with the feature selection techniques. A pairwise correlation matrix was computed to remove the highly correlated predictors, as having these predictors in the analysis can increase the bias as well as degrade the interpretability of the model. After removing the features with the highest pairwise correlation, the XGBoost classifier was used to construct the relative feature importance by constructing boosted decision trees within the model. In the end, the ExtraTreesClassifier was used to aggregate the results of multiple decorrelated decision trees to generate feature importance. After this pipeline was implemented, the top two nutrients were selected, which were sensed and regulated using an IoT set-up that is described in detail in the next section.

### 2.4. IoT System for Nutrient Regulation

In order to build the IoT system for regulating nutrients, the entire system was divided into three parts, namely: (a) the sensor subsystem, (b) the feedback loop and (c) the actuator system. The sensor subsystem consists of two sensors, manufactured by Vernier, to measure the important nutrients and output the data to the Raspberry Pi through USB.

The sensors were selected by a thorough trade study, with consideration of cost, level of accuracy, type of interface required and ease of use. The two Vernier sensors which were used can measure calcium and ammonium up to 40,000 parts per million (ppm), with an accuracy of ±10%. They have the capability to interface with a Raspberry Pi through USB or Bluetooth, and are programmed through Python using the manufacturer’s Python driver. These sensors are calibrated by rinsing with distilled water. Each sensor samples the water five times before outputting an average of the results to the feedback loop which computes the values in ppm.

To properly measure and regulate the nutrient levels within the entire system, a feedback loop is used to continually monitor the environment. It connects to both the sensor system and actuator system to retrieve current nutrient amounts and release each nutrient in uniform concentration if needed. This program was created using the Python programming language due to the availability of libraries developed for usage with the Vernier ion-selective electrodes. The sensors themselves do not have any form of serial communication, so USB connectivity was used. A Python-based feedback loop also presents the opportunity to develop highly object-oriented code for ease of debugging and future improvements. A Raspberry Pi Model 3B+ was selected to run the program. This device also contains multiple USB connections to power the sensor module.

In order to run, the feedback loop first initializes all connected systems including the Vernier ISE probes and actuators. Upon a successful set-up sequence, the loop begins to take readings from the Vernier sensors and averages them five times to use as the current nutrient level. If the measured levels remain below the targeted nutrient levels, the actuator system receives a signal through the GPIO pins of the Raspberry Pi to run one cycle of nutrient release, slightly increasing the concentration of the given nutrient. As the nutrient deficit is not being absolutely compared, the feedback loop’s iterative cycling helps to continually maintain a target goal. Rather than dropping an estimated amount in all at once to make up any measured deficit, the continual cycling and addition of small amounts better approaches a consistent system with a smaller chance of nutrient oversaturation or undersaturation.

The actuator subsystem in this case consists of a PIC microcontroller, 12 V tolerance motor modules and two 12 V stepper motors to dispense the nutrients. The stepper motors are connected to the motor modules which supply the motors with 12 V as well as the signals to rotate the dispensing portion. The motor modules are then connected to the PIC microcontroller, which determines the rotational direction of the motor and the speed of rotation. The motors are activated when the PIC microcontroller receives a bit signal from the Raspberry Pi to indicate that more nutrients are needed.

## 3. Results

**Figure 3:**
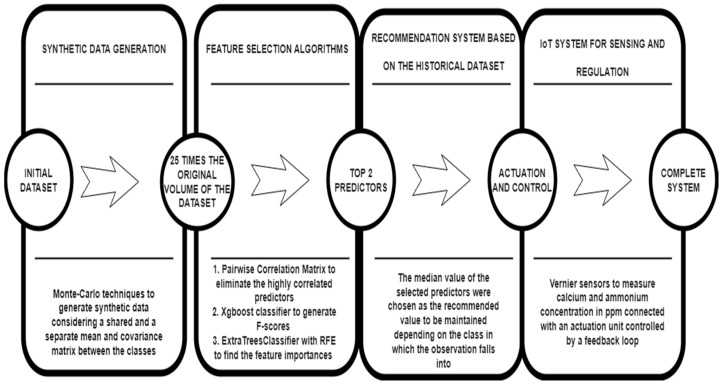
Pipeline of the complete system for sensing and actuation of nutrients for coupled systems in commercial aquaponic set-ups.

A brief overview of the pipeline used to carry out the analysis is described in Figure 3 below.

The first step before analyzing the dataset is to remove the predictors which had zero or null variance in their values. Carbonate was removed from the dataset as it had a value of zero throughout the dataset. After this, it was decided to proceed with synthetic data generation using Monte-Carlo approaches. Standardization or normalization of the data points was not carried out as all the predictors were distributed normally. A total of 2675 data points were generated for each class using a variation of the Monte-Carlo technique in which a separate mean and covariance matrix was produced for each class. Similarly, 5350 synthetic data points were generated, sharing the mean and covariance matrix between both the classes. This resulted in a total of 10,700 observations with 12 predictors, which were then used for further analysis. As the process of synthetic data generation and feature selection algorithms was moderately computationally exhaustive, an x64-based Intel(R) Core(™) i7-processor-based system with 8 GB RAM was used to run the algorithms.

**Figure 4:**
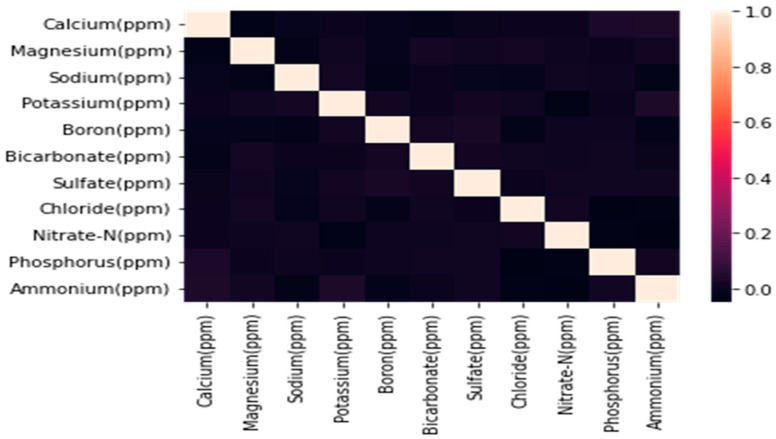
Pairwise correlation matrix among all the nutrient predictors in the dataset.

As mentioned in the section above, a pipeline of feature selection techniques was applied to the dataset to select the top two nutrients. At first, a pairwise correlation matrix was generated between the predictors and is mentioned in Figure 4.

**Figure 5:**
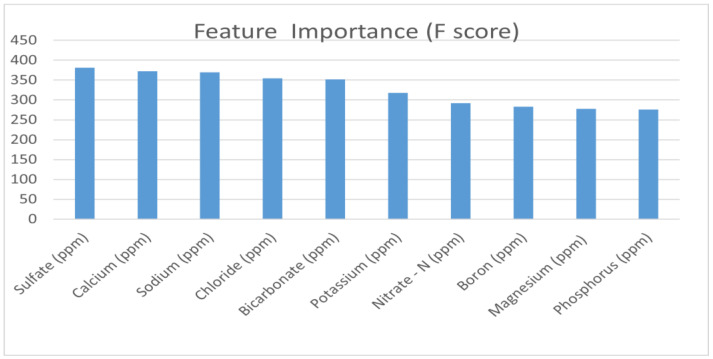
F-score of each predictor generated by the XGBoost algorithm.

From Figure 4, it can be inferred that the pairwise correlation between the predictors is not greater than 0.05. Therefore, it was decided to proceed with the entire dataset without eliminating any predictors. Further, the XGBoost algorithm was used to generate F-scores for each predictor, as shown in Figure 5.

Figure 5 shows that the feature importance of sulfate is the highest, as the F-score generated by the XGBoost algorithm is around 375. This was followed by the feature importance of calcium, sodium, chloride, bicarbonate and potassium, with values ranging between 325 and 375. The rest of the nutrient predictors were excluded from our analysis before proceeding to the third step of the feature selection pipeline.

**Figure 6:**
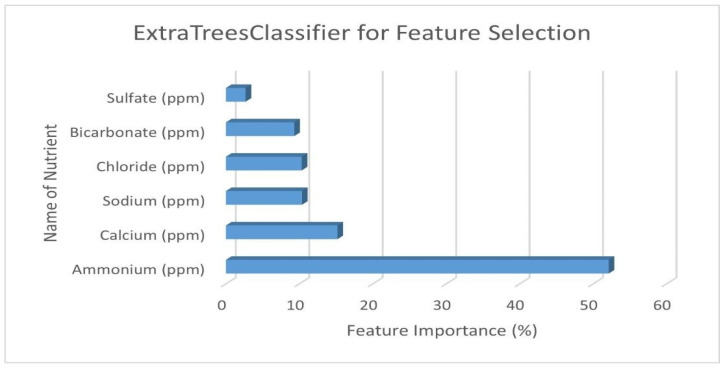
Feature importance of each predictor generated by the ExtraTreesClassifier.

The final step in the feature selection process is applying the ExtraTreesClassifier with Recursive Feature Elimination on the trimmed dataset to generate feature importance, as shown in Figure 6.

**Figure 7:**
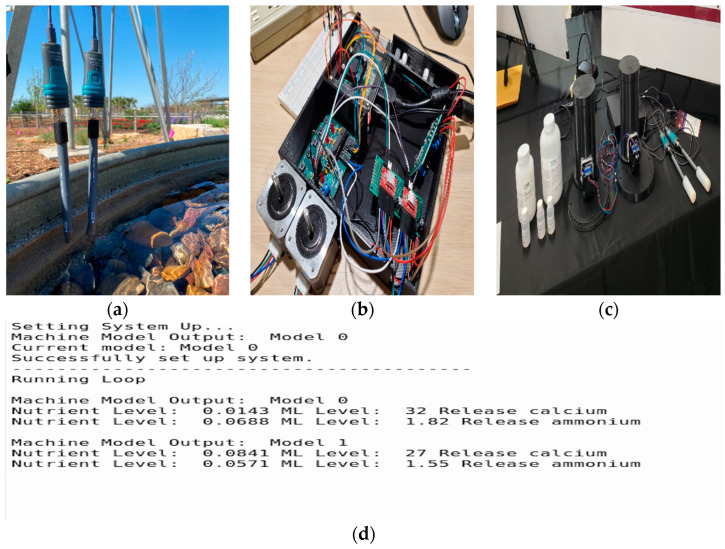
(**a**). A set-up of the Vernier sensors to measure calcium and ammonium; (**b**). a set-up of the motor control units and the MCUs to control the actuators; (**c**). a prototype of the full system showing the nutrients with the actuator units used to carry out their dispensation and (**d**). a feedback loop output communicating between the sensor and actuator modules.

The ExtraTreesClassifier revealed that the feature importance values of ammonium and calcium were 52% and 16%, respectively, making up 68% of the total feature importance in the dataset. Next, the main focus was to automate the sensing and actuation of these nutrients using a feedback loop. As discussed above, two Vernier sensors were used to measure ammonium and calcium, and two actuator modules were designed to release these nutrients if they fall below the recommended concentrations. A model set-up of the sensing, actuation and feedback unit is shown in Figure 7.

From Figure 7, it was inferred that the output of the ML model, which is either 0 or 1, states the month in which the observations were recorded and has a set of recommended levels of nutrients that need to be maintained in the aquaponic solution. For each recommendation value stated in the table, the median of the nutrient values from the historical dataset per class was chosen as the concentration that should be maintained in the aquaponic solution.

**Figure 8:**
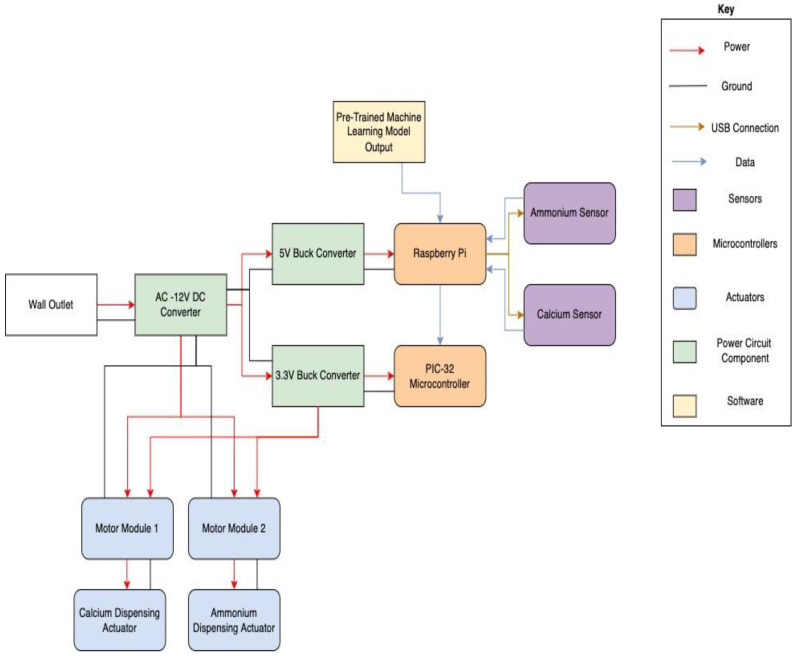
Detailed block diagram of the IoT-based sensing and dispensing system.

The overall nutrient regulation and monitoring system functions through the combination of three main components and is explained in detail with the help of the block diagram in Figure 8.

The first subsystem involves the nutrient level input to be fed through the rest of the system. In order to measure the current level of calcium and ammonium in the water at any point in time, two Vernier ion-selective electrodes were inserted into the water as shown in Figure 7a and given at least 30 min to accumulate to the present concentration. The sensors then communicated the measured nutrient concentration data through USB protocol with the Raspberry Pi 3 B+ that is situated in the 3D printed box displayed in Figure 7b.

The second main component of the nutrient and regulation system involved the feedback loop which connects both the sensor and actuator systems. The Raspberry Pi ran this Python-based program to take in sensor readings and output signals for nutrient dispensing, as shown in Figure 7d. In order to integrate with the Vernier sensors, the program imported the GDX library provided by Vernier which holds initialization and interaction functions. With the sensors able to be communicated with, the feedback loop was able to call the sensors five times at an interval of five min in order to attain an average measured nutrient level to use. This level (in ppm) was then compared to the target nutrient levels defined by one of the two models listed in Table 1. The current Machine Learning model being used was altered manually through the use of a GPIO toggle switch depending on the season in which the crops were grown. Another switch was used as well to represent a pause function that allows the user to stop any function in order to perform side tasks such as sensor calibration while preventing unnecessary nutrient dispersal. If the current amount of each nutrient was lower than expected, a signal was sent through GPIO pins on the Raspberry Pi to the PIC32 microcontroller connected to the actuators.

The final main component of this system involved the Nema 17 stepper motor actuators which dispensed the powdered nutrients. This system only operates when a high signal is received from the Raspberry Pi to the PIC32 microcontroller. The PIC32 interprets this signal to initiate the stepper motors 90 degrees. These actuators would spin a perforated wheel connected to a tank filled with each nutrient, as seen in Figure 7c. The tanks themselves had holes cut out at the bottom which line up with holes inside of the connected wheel. After the actuator was able to complete a cycle, a filled wheel hole was able to dispense 0.6 g of the respective powder into the tank of water. This cycle continued until the nutrient levels reached a stable level.

A recommendation system which has been implemented on the basis of the binary data-driven model is shown in Table 1.

Based on the above set of recommendations, some test runs were carried out and the results of the IoT-based system using Machine Learning as a Decision Support System are presented in detail in Table 2 below.

In order to demonstrate the efficiency of the proposed aquaponic system, several test runs were carried out over a period of five months from the end of October 2021 to early March 2022 in an aquaponic system containing 450 gallons, and the results of the test runs are presented in Table 2.

Some of the observations which are clearly demonstrated from Table 2 are as follows. As the sensors operate five times in a single cycle and can be seen in the test run dated 22 October 2021, the amount of ammonium sulfate dispensed in the first iteration increases the concentration of ammonium in the aquaponic solution by 0.29 ppm. This cycle of sensing and dispensing the above stated nutrients happens until both the concentrations of ammonium and calcium reach the desired level as stated in Table 1. The calcium nitrate and ammonium sulfate powders are released in dosage of 0.5 g and the respective nutrient concentrations are measured every cycle. This has been further demonstrated in the last three test runs on 9 December 2021, 8 January 2022 and 3 February 2022 where the sensing and actuation of nutrients do not run for five cycles as the desired concentration of the nutrients is achieved much before that. As the system monitors the nutrients in a closed loop, the loop is terminated before hitting five cycles.

## 4. Discussion

The main motivation behind developing this approach is to build a recommendation system with real-time sensing and actuation units to regulate nutrient levels in the aquaponic solution for optimal growth of fish and crop plants in a single set-up. In order to achieve this, the prerecorded observations from the historical dataset were used to design the approach and a set of rules was prescribed for both the classes.

This is the first of its kind where IoT systems were combined with Machine Learning for optimizing nutrient supply in aquaponic solutions. The major advantage of a data-driven IoT system compared to a traditional aquaponic system is cost savings through improved yield and quality of produce via the integration of AI. This in turn significantly increases the profits generated from the aquaponic operations. Another solution provided in this research is the reduction in the amount of nutrients supplied for plant growth. In many of the earlier aquaponic studies, excess phosphorus accumulation due to unregulated use of fertilizers has resulted in eutrophication, leading to algal growth and a steep decline in fish populations [36]. The nutrient accumulation problem has been addressed in this research through IoT-based nutrient optimization.

For the observations for which the month class is 0, cultivation occurs during the dry and hot summers of Texas. During these months, the fish in the aquaponic set-up grew from fries to the adult stage. As ammonium is a byproduct formed from protein metabolism, it is excreted in high amounts during these months [37,38]. Since high ammonium concentrations are toxic to fish, it is important to restrict ammonia to a maximum level of 1.82 ppm. On the other hand, ammonium is a source of nitrogen for plants [39], resulting in higher yields, though greater than optimal levels can also be injurious to crop growth. For the observations for which the month class is 1, cultivation is performed during the cold winter months. During this period, the fish growth is minimal, and in turn the amount of ammonium released in water is comparatively low. The concentration of ammonium during the winter months can be restricted to 1.55 ppm, which is sufficient for plant growth and is also a safe limit for sustainable fish growth in commercial set-ups.

Most of the fish grown during the summer months in aquaponic set-ups in Texas are warm-water fish species. As mentioned above, calcium is an important element required for normal growth and reproduction of fish species [40]. Calcium levels need to be maintained at 32 ppm during these months, which allows for maintenance of water hardness between 75 and 125 ppm, while still being safe for plant growth. Calcium concentrations beyond this level can cause tip burn in lettuce plants. During the winter months, plant growth is considerably slower and excess levels of carbonates or bicarbonates can be toxic to plants [41]. Therefore, the recommended level of calcium in the aquaponic solution was 27 ppm, which was sufficient to sustain fish growth without injuring the winter crops.

This study specifically addresses the optimization of the top two nutrients (ammonium and calcium) which should be regulated depending on the month in which the plants and fish are grown in a closed-loop control aquaponic set-up. As ammonium is a key factor in controlling the pH and TDS of the solution, regulating the ammonium concentration addresses most of the issues which may occur due to TDS imbalance in the solution [42,43]. On the other hand, the concentration of calcium plays an important role in determining the hardness of the solution. Therefore, regulating both of these nutrients through an IoT-based sensing and actuation system ensures that pH is adequately regulated for promoting optimal growth and yield of both fish and crop plants in a single set-up.

As discussed earlier, multiple tests were carried out between October of 2021 and February of 2022 to validate our design in a leading commercial aquaponic set-up in Grimes, TX. Instead of randomly adding nutrients to the aquaponic set-up in varying concentrations as in traditional decoupled aquaponic set-ups, nutrients were added in small quantities and were regularly monitored using nutrient sensors specified before. The biggest advantage of this automated system is reduction in the overall cost incurred in cleaning up the unregulated aquaponic systems due to eutrophication resulting from overaccumulation of nutrients. The other advantage of the system is reduction in the number of nutrients which are monitored in commercial aquaponic systems. Barring the increase in yield and size of the produce which has been discussed in the conclusion, these are the major advantages which have been observed in our controlled aquaponic set-up compared to that of the existing commercial operations.

In most commercial aquaponic systems, decoupled systems are used where fish and plants are not connected together. Our system goes on to present a coupled system for growing plants and fish in a single set-up. In general, tap water is used as the source of water supply in most of these decoupled systems, with deregulated addition of nutrients such as iron, calcium, etc., and the growth of plants and fish is observed in individual set-ups. The major improvements which we suggest in this research are coupling both the systems together, which reduces the cost of the system significantly and promotes the recyclability of the water with routine sensing, and addition of nutrients based on the month lettuce was cultivated. The concern regarding monitoring of heavy metals is to be addressed in the future, for which we are working on building a spectrometer from scratch which can help in real-time monitoring of the heavy metals in the aquaponic solution.

## 5. Conclusions and Future Work

Using the dataset recorded from three aquaponic farms in Southeast Texas, the two most important predictors identified from the existing design, after generating synthetic data and carrying out dimensionality reduction, were the concentrations of calcium and ammonium. These two nutrients were regulated using an IoT-based sensing and actuation system in a closed-loop set-up. The tests were carried out for the cycle of 21 days to grow romaine lettuce and were verified experimentally as to how the proposed recommendations positively impacted plant and fish growth in a single system. The size and yield of the lettuce were compared to the ones grown in unregulated aquaponic environments and the yield showed significant increase in size, with some of them being as large as 40 to 45 inches in diameter. The cost involved in regulating nutrient parameters compared to traditional aquaponic environments also decreased by more than 75%.

In the future, a cloud database can be set up to host the Machine Learning model and store data dynamically. The size of the sensing and actuation modules described above can be scaled up to regulate more macronutrients and heavy metals to build a comprehensive unit to be installed at commercial set-ups. The data collected in our case to generate the AI-based approach were collected from three aquaponic farms in Texas where the terrain is mostly flat. More data can be added in the future from diverse geographical locations with more variable weather conditions to improve the robustness of the model.

As all the data on which our model was trained to build an AI-based approach were recorded from aquaponic farms located in Texas, the IoT system developed here cannot be used directly in places that are extremely hot or cold. The important features determined by our dimensionality reduction techniques are bound to change in those conditions due to high variance expected in the dataset. However, this study provides a general approach that can be adapted to a range of environments and aquaponic settings with adequate modifications. Future research should investigate the effectiveness of the IoT approach for optimizing nutrient supply in extreme climatic conditions and diverse management scenarios.

## Figures and Tables

**Table 1:** The recommendation system for nutrient regulation in aquaponic environments.

Sl. No.	Class Name	Calcium Concentration (ppm)	Ammonium Concentration (ppm)
1	0 (April to October)	32	1.82
2	1 (November to March)	27	1.55

**Table 2:** Test run to demonstrate control and actuation of nutrients in the IoT set-up.

Date	Iteration No.	Measured Ammonium Concentration (ppm)	Amount of Ammonium Sulfate Added	Measured Calcium Concentration (ppm)	Amount of Calcium Nitrate Added
22 October 2021	1	1.1	0.5 g	29.7	0.5 g
	2	1.39	0.5 g	31.1	0.5 g
	3	1.65	0	31.41	0.5 g
	4	1.65	0	31.7	0.5 g
	5	1.65	0	32.1	0
10 November 2021	1	1.4	0.5 g	30.2	0.5 g
	2	1.68	0	30.5	0.5 g
	3	1.68	0	31.2	0.5 g
	4	1.68	0	31.57	0.5 g
	5	1.68	0	32.3	0
9 December 2021	1	1.32	0.5 g	32.8	0
	2	1.6	0	32.8	0
8 January 2022	1	1	0.5 g	31.8	0.5 g
	2	1.3	0.5 g	32.14	0.5 g
	3	1.6	0 g	32.5	0 g
3 February 2022	1	1.4	0.5 g	32.3	0 g
	2	1.72	0	32.3	0 g

## Data Availability

The dataset used in this case has been described in detail in the sections above. The researchers would be willing to provide more details if needed.
